# Construction Notes

**Published:** 1895-04-27

**Authors:** 


### CONSTRUCTION NOTES.

### New Infirmary, Paisley.

Plans have been prepared for this proposed infirmary by Mr. T. G. Abercrombie, architect, Paisley. The building will consist of two storeys and attics, the central block and portions of the wings jutting slightly out from the main block. In the centre of the building is to be the "administrative" block, with the principal entrance. The east wing will contain circular wards, while that on the west will be devoted to rooms for sick nurses on the ground floor and doctors' bedrooms above. The two circular wards of 12 beds each are to be allotted to 12 boys and girls respectively, with accompanying duty-rooms, day-rooms, and bath-rooms. Between the pavilions spaces will be kept as airing grounds for the patients. The administrative block will contain vestibule and hall, porters, visitors, and doctors' rooms, the board-room and committee-room being on the first floor. The matron's rooms are situated on the ground floor, and above will be the operating theatre, anæsthetising-room, and clinical laboratory. Kitchens and servants' rooms are on the top floor of the building. Very special attention has been given to the arrangements for heating and ventilation. The estimated cost of the building is £6,000. In addition to the hospital, a nurses' home (the gift of Mr. Peter Coats) is being erected in the grounds.

### New Infirmary, Southport.

This handsome new building is situated in the Scarisbrick New Road, and is of commanding appearance. The entrance into the infirmary is by a large front stone doorway, which leads to the outer vestibule. Beyond this department there is another vestibule, from which entrance is gained to the main hall. On the right is the porter's room, the secretary's office, and board-room, the latter two being directly connected. On the left are the matron's sitting-room, visitors' waiting-room, and the house surgeon's sitting-room. From this main hall stairs lead up to the nurses' abode, which occupies the first floor of the central block and contains the following: Nurses' sitting-room, matron's bed-room, sixteen separate bed-rooms fo: the nurses, linen and housemaid's rooms, box-room, &c. An entire suite of rooms is arranged for the doctors, which are arrived at from the house surgeon's sitting-room; direct communication is given to the accident receiving-room. The operating-room is provided with special floor arranged by Messrs. Patteson, of Manchester. From the centre of the corridor where the operating-room is situated, another corridor of similar proportions branches off. On its left or western side are all rooms that are connected with the accident receiving and out-patient department. The dispensary is centrally situate in the main corridor. The kitchen offices are complete in every detail, and on the same extensive scale as are all the other arrangements of this most excellently-constructed building. The children's department is a complete little hospital of itself, entirely isolated from the rest of the building. This, again, contains all kitchen arrangements and laundry accommodation. The ward is circular in construction, and has 12 beds. The men's ward is at the west end of the main building, the women's at the eastern end. Each wing is fitted with windows on either side for the purpose of cross-ventilation. French windows lead down into the gardens, which, being of great extent, form a park-like surrounding to the infirmary. Lavatories, sinks, bath-rooms, and every convenience are added to the various corridors. A separate building to the north of the kitchen block contains a boiler-house, a coal-house, disinfectant chamber, soiled linen and sorting rooms, &c. Further on, towards the rear, is the mortuary chamber, with every necessary accessory thereto. The three main wards are connected by telephone, and hot water is supplied to almost every room. The whole contract was placed in the hands of Messrs. Wishart and Irving, of Southport.

